# KIT is required for hepatic function during mouse post-natal development

**DOI:** 10.1186/1471-213X-7-81

**Published:** 2007-07-05

**Authors:** Laetitia Magnol, Marie-Clémence Chevallier, Valérie Nalesso, Stéphanie Retif, Helmut Fuchs, Martina Klempt, Patricia Pereira, Michel Riottot, Sandra Andrzejewski, Bich-Thuy Doan, Jean-Jacques Panthier, Anne Puech, Jean-Claude Beloeil, Martin Hrabe de Angelis, Yann Hérault

**Affiliations:** 1 Institut de Transgénose, TAAM, UPS44, IEM UMR6218, CNRS, Université Orléans, rue de la Férollerie Orléans, France; 2 GSF Research centre, Institute of Experimental Genetics, Ingolstaedter Landstrasse Neuherberg, Germany; 3 NOPA, INRA-Université Paris Sud, Orsay, France; 4 CNRG-CNG Evry, Rue G. Crémieux, Evry, France; 5 CBM CNRS, Rue Charles-Sadron, Orléans, France; 6 UMR955 INRA-ENVA Maisons-Alfort, Avenue du Général de Gaulle, Maisons-Alfort, France

## Abstract

**Background:**

The *Kit *gene encodes a receptor tyrosine kinase involved in various biological processes including melanogenesis, hematopoiesis and gametogenesis in mice and human. A large number of *Kit *mutants has been described so far showing the pleiotropic phenotypes associated with partial loss-of-function of the gene. Hypomorphic mutations can induce a light coat color phenotype while complete lack of KIT function interferes with embryogenesis. Interestingly several intermediate hypomorphic mutations induced in addition growth retardation and post-natal mortality.

**Results:**

In this report we investigated the post-natal role of *Kit *by using a panel of chemically-induced hypomorphic mutations recently isolated in the mouse. We found that, in addition to the classical phenotypes, mutations of *Kit *induced juvenile steatosis, associated with the downregulation of the three genes, *VldlR*, *Lpin1 *and *Lpl*, controlling lipid metabolism in the post-natal liver. Hence, *Kit *loss-of-functions mimicked the inactivation of genes controlling the hepatic metabolism of triglycerides, the major source of energy from maternal milk, leading to growth and viability defects during neonatal development.

**Conclusion:**

This is a first report involving KIT in the control of lipid metabolism in neonates and opening new perspectives for understanding juvenile steatosis. Moreover, it reinforces the role of Kit during development of the liver and underscores the caution that should be exerted in using KIT inhibitors during anti-cancer treatment.

## Background

KIT is a well-known receptor tyrosine kinase controlling melanogenesis, hematopoiesis, gametogenesis, hearing, mast cells and the survival of Cajal cells from the intestine [1-7]. Furthermore Kit is expressed in several epithelial endothelium or endocrine cells but also in cholangiocytes during development and in the adult [8,9]. The binding of KITL, induces the dimerization of the KIT molecule, followed by a change in the configuration of the intracellular domain and the autophosphorylation of the receptor, opening several docking sites for signal transduction molecules containing SH2 binding domains. Hence the KIT signal is transduced by several pathways including that of the SRC family kinases, PLC-γ, Ras/MAPK, PI3K-AKT-mTor, JAK/STAT and also through the activation of the SNAI2 and MITF transcription factors [for review see [10-12]]. KIT belongs to the class III of the receptor tyrosine kinase family with CSF1R, PDGFR-a,-b, KDR, FLT-1, -3 and -4, characterized by the presence of an autoinhibitory juxtamembrane (JM) domain. Without phosphorylation, the JM domain is inserted in between the N- and C-lobes blocking the DFG (Asp-Phe-Gly) conserved motif of the activation loop in an inactive conformation. Upon phosphorylation, changes in the conformation of the JM domain opens up the ATP binding site and repositions correctly the activation loop in the kinase domain [13,14]. Mutations that affect the activation loop are found in human mastocytose or leukemia, while deletions or insertions in the JM domain are observed in gastro intestinal tumor (GIST) and often correspond to gain-of-function [15]. The treatment of the *Kit*-induced tumors had taken a promising perspective with the use of STI-571 (Imatinib, Gleevec Novartis, Basel, Switzerland), that binds to the ATP-binding site, stabilizing the autoinhibitory structure with an inactive tyrosine kinase domain [14].

In human, *Kit *loss-of-function is linked to Piebald trait [16,17] and a large panel of mutations is reported [for a synthetic panel see [18]]. Similar pigmentation defects are observed in mouse models [2] and the analysis of KIT functions was facilitated by a large number of hypomorphic murine mutants [4,12]. Some correspond to point mutations, others to large chromosomal rearrangements obtained as spontaneous or induced by chemicals or radiation, while a few were engineered [2,8,19,20]. Complete lack of *Kit *interferes with post-natal viability [8] while hypomorphic mutations could induce not only a variable degree of defects during melanogenesis, hematopoieisis and gametogenesis but also growth retardation and juvenile mortality [4,18].

In this report we used a panel of mutants isolated from genome-wide mutagenesis screens to investigate the post-natal role of KIT. These mutations correspond to a new allelic series of hypomorphic *Kit *alleles that alter differentially the melanogenesis, hematopoiesis and gametogenesis. In two such mutants, we observed an impaired viability of the homozygotes before weaning. Clearly, growth retardation and premature death of neonates in both *Kit *mutants were associated with juvenile steatosis during the suckling period. Further analysis pointed out that *Kit *is expressed in the post-natal liver and *Kit *mutants were suffering from an early defect in triglycerides (TG) export from the liver correlated with the downregulation of key genes participating in the control of hepatic lipid metabolism. This series of evidence established the major role of Kit in controlling liver triglycerides metabolism in neonates.

## Results

### Characterization of a new allelic series of mutants affecting the *Kit *gene

**Figure 1:**
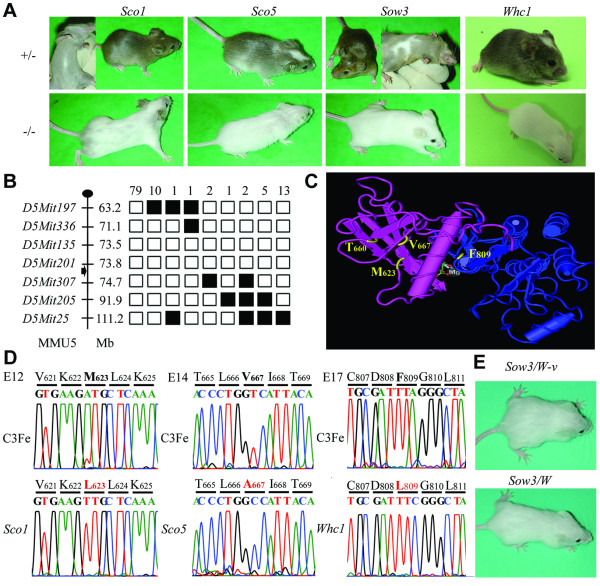
**New ENU-induced alleles at the *Kit *locus**. (**A**) Heterozygous individuals carrying the *Sco1*, *Sco5*, *Sow3 *and *Whc1 *mutations display white spotting on the belly and a white forehead blaze while homozygotes (-/-) have a white coat color with black eyes. (**B**) Haplotype analysis of 114*Sow3/+ *mutant mice derived from the F1C3FeB6 × B6 strategy. Markers are shown with their position (Mb) from the centromere of chromosome 5 (position based on Ensembl v33) and the position of the *Kit *gene is shown by an arrowhead. The black and white boxes represent respectively the B6 and the C3Fe alleles. The cumulative number of mice sharing the same haploid genotype is noted at the top of each column. (**C**) The mutated amino-acids found in the *Sco1*, *Sco5 *alleles are located in the N-terminal hinge part of the intracellular tyrosine kinase domain of the KIT protein, at the vicinity of the T660 amino acid, mutated in the *Kit*^*W*-*v *^allele, whereas the F809 modified in *Whc1 *is found at the level of the Mg^2+ ^binding loop of the activation segment. (**D**) Sequence chromatograph spanning the *Sco1*, *Sco5 *and *Whc1 *mutation sites respectively in exons 12, 14 and 17 of the *Kit *gene compared with that of a wild-type control from the same genetic background (C3Fe). The amino-acid substitution is labeled in red. (**E**) Transheterozygous animals carrying the *Sow3 *and the *Kit*^*W*-*v *^or the *Kit*^*W *^allele exhibit a white coat color and pigmented eyes.

To analyze the role of *Kit *in neonates, we used new semi-dominant ENU-induced mutant mouse lines named *Sco1*, *Sco5 ("Spotted coat")*, *Sow3 *("*Sister of W"*) and *Whc1 *("*White coat"*), displaying white head blazes and belly spots, that were isolated in genome wide mutagenesis programs [21,22] (Figure 1A). The corresponding mutations were mapped on the mouse chromosome 5 near the *Kit *locus (Figure 1B; data not shown). With the sequencing of the *Kit *gene from the *Sco1*, *Sco5 *and *Whc1 *homozygotes, unique missense mutations were found in the intracellular tyrosine kinase domain that affect M623L for *Sco1*, V667A for *Sco5 *and F809L for *Whc1 *(Figure 1C–1D). These changes were absent in the original C3HeB/FeJ and other strains. No mutation was found in the exonic and the splice junction sequences of the *Kit *gene from the *Sow3 *mouse mutant line, suggesting that *Sow3 *affects the regulation of the *Kit *gene as described in other mouse mutants [19,23,24]. Nonetheless, a series of complementation assays with other known alleles including *Kit*^*W*^, *Kit*^*W*-*v *^and *Kit*^*tm*1*Alf *^confirmed that Kit function was altered in *Sow3 *(Figure 1E). Therefore, the data support that the mutants described here represent a new allelic series at the *Kit *locus, with three missense mutations (*Sco1*, *Sco5 *and *Whc1*) and one probable mutation affecting the regulation of the *Kit *gene (*Sow3*).

**Figure 2:**
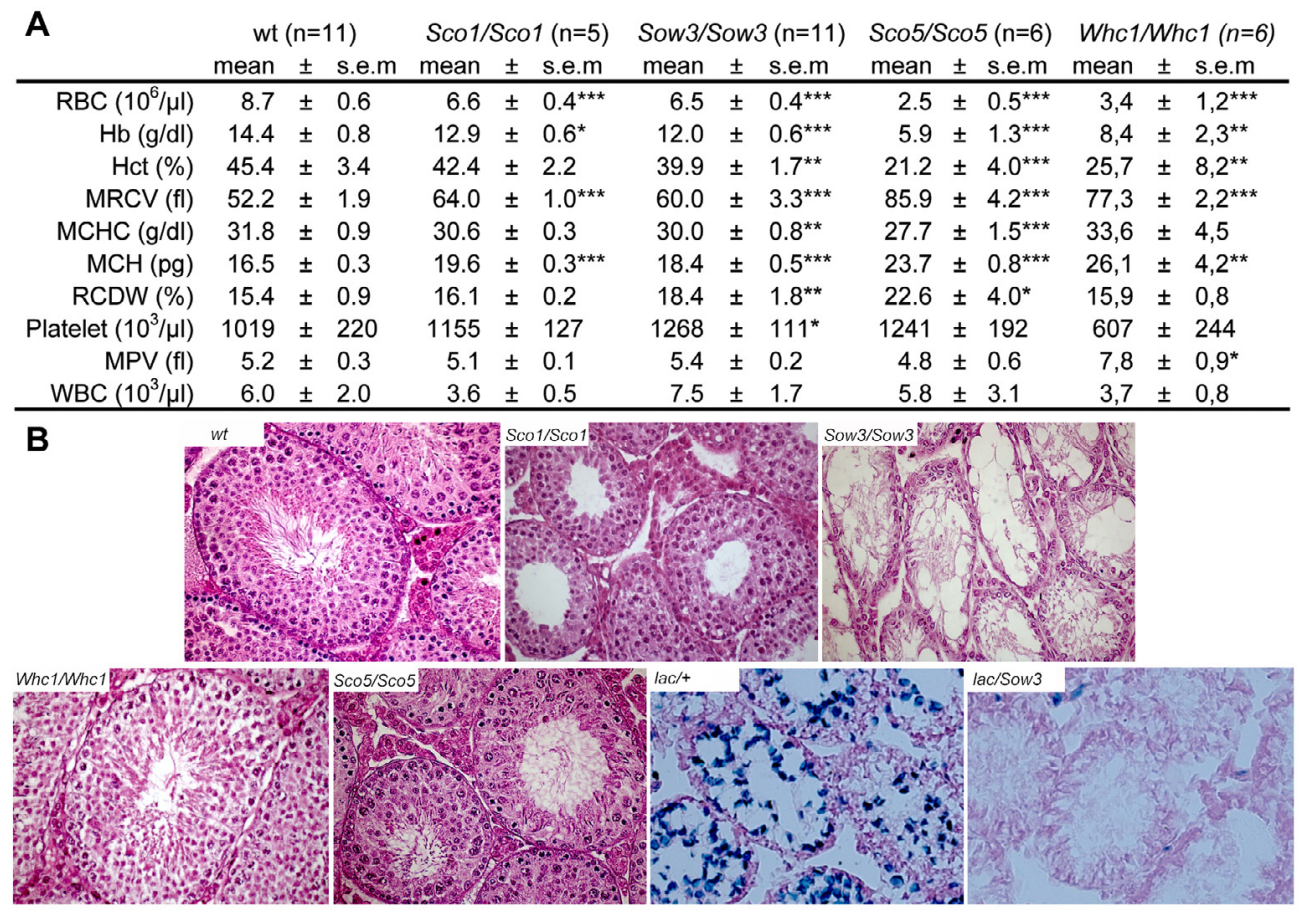
**Blood and germ cells phenotypes in mice homozygous for the *Sco1*, *Sco5 *and *Sow3 *alleles**. (**A**) Specific parameters including Red Blood Cells (RBC), Hemoglobin (Hb), hematocrit (Hct), Mean Red Cell volume (MRCV), Mean Cell Haemoglobin Concentration (MCHC), Mean Cell Hemoglobin (MCH), Red Cell Distribution Width (RCDW), Platelet, Mean Platelet Volume (MPV) and White Blood Cells (WBC) were determined for homozygous males (*Sco1/Sco1*, n = 5; *Sow3*/*Sow3*, n = 11; *Sco5*/*Sco5*, n = 6; *Whc1*/*Whc1*, n = 6) and compared to wild-type (wt, n = 11) individuals. Results are expressed as mean ± sem. (**B**) Testis were sectioned and stained with Hematoxylin and Eosin showing a normal appearance and organization in the *Sco1 *and *Sco5 *mutants. On the contrary, the seminiferous tubules of *Sow3 *adult mutant individuals are lacunar with a complete absence of all the layers corresponding to the differentiation of spermatogonial stem cells. The β-galactosidase pattern of expression reveals that *Kit*^*tm*1*Alf*/*Sow*3 ^mutant mice showed no pattern of spermatogenesis compared to *Kit*^*tm*1*Alf*/+^.

Homozygous animals generated for the four mutations displayed similar white coat pigmentation with black eyes (Figure 1A). However, *Sco1 *homozygotes showed a few grey hairs and pigmented ears suggesting that this allele alters later stages of pigmentation [25]. Blood analysis showed changes characteristic of macrocytic anemia, with a significant reduction of hematocrit and red blood cell count, together with an increase in mean red cell volume and in mean cell hemoglobin content for all the adult mutant mice (Figure 2A). Hematopoietic defects were more severe in the *Sco5 *and *Whc1 *homozygotes than in *Sow3 *and *Sco1 *mutants with however some little variations between males and females. Finally, histological analysis of testis isolated from adult males homozygous for *Sco1*, *Sco5 *and *Whc1 *showed different stages of germ cells differentiation while *Sow3 *homozygotes revealed a complete lack of spermatogenesis (Figure 2B). These results were confirmed by the absence of β-galactosidase staining in the seminiferous tubules of *Kit*^*tm*1*Alf*/*Sow*3 ^transheterozygotes (Figure 2B) and assessed by a fertility test. Only the *Sco1 *and *Sco5 *homozygotes were able to reproduce but no progeny was ever obtained from crosses between *Sow3/Sow3 *males and wild-type females (data not shown). Thus, *Sco1*, *Sco5*, *Sow3 *and *Whc1 *are hypomorphic mutations of *Kit*, with the expected effects, although variable, on melanogenesis, hematopoiesis and gametogenesis.

### *Sco5 *mutant analysis unravels a function of *Kit *during post-natal development of the liver

**Figure 3:**
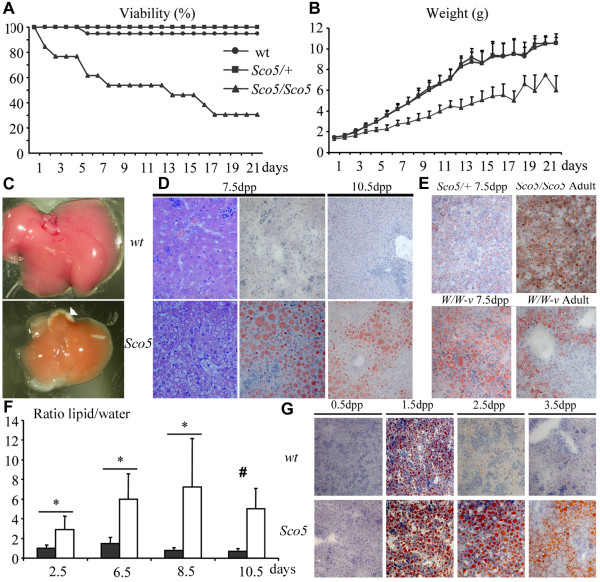
**Growth and liver defects in *Kit*^*Sco*5 ^homozygotes during post-natal development**. (**A**) Viability and (**B**) body weight curves of *Sco5/Sco5 *individuals generated from heterozygous *Sco5/+ *intercrosses (*Sco5/Sco5 *n = 13; *Sco5/+ *n = 28; wt n = 16) reveal that mutant homozygotes are strongly affected during post-natal development compared to control littermates. (**C**) At 7.5 dpc, livers of mutants have a more yellowish color which was associated with the appearance of some white areas (white arrowhead). These alterations were never observed in wild-type (wt) littermates. (**D**) Hematoxylin and eosin, and Oil-red-O stainings reveal the swelling of hepatocytes with formation of large lipid-containing vesicles in *Sco5 *homozygotes versus wild-type mouse littermates at 7.5 and 10.5 dpp (magnification ×40). (**E**) Staining of *Sco5/+ *individuals at 7.5 dpp shows a relative increase in lipid droplets inside hepatocytes compared to wild-type (**D**). A similar defect is observed in the liver of 7.5 dpp old mice, surviving adult *Sco5 *homozygotes and *W/W-v *compound *Kit *mutants. (**F**) The lipid accumulation in the liver was studied by NMR during post-natal development at various ages on wt (n = 5; black) and mutant (n = 9,8,8,3; white) individuals. No statistical analysis was possible at 10.5 dpp because of the death of 6 mutants out of 9 (#), but the lipid/water ratio is still increased in mutant mice, at 10.5 dpp, in coherence with mortality scale and histology. (**G**) At 0.5 dpp, the Oil-red-O staining of liver sections from *Sco5 *homozygous and control mice which had not yet suckled, are identical showing no accumulation of lipids. However, after breast feeding, hepatocytes from control newborns appear to stock lipids inside microvesicles at 1.5 dpp, a phenomenon that is not observed at later stages. On the contrary, in mutant mice which have suckled milk from their mother, lipids accumulation is still found in hepatic cells after 1.5 dpp.

An impaired viability affected *Kit*^*Sco*5 ^homozygotes before weaning, with only 21 mutants recovered out of 241 offspring from *Kit*^*Sco*5/+ ^intercrosses. Although *Sco5*/*Sco5 *genotypes scored at birth with a Mendelian ratio, almost 50% of them died before 7.5 days post partum (dpp), and only 25% survived at weaning (Figure 3A). In addition, *Kit*^*Sco*5/*Sco*5 ^homozygotes displayed a growth defect at 3.5 dpp, with a weight difference reaching up to 50% of the control at 21 dpp (Figure 3B). Comparable phenotypes were also noted in *Kit*^*Whc*1/*Whc*1 ^and *Kit*^*W*/*W*-*v *^animals, thus we analyzed further this post-natal lethal phenotype.

Necropsy analysis revealed that the liver from *Kit*^*Sco*5/*Sco*5 ^animals at 7.5 dpp had a tan yellowish color (Figure 3C) although it had a normal relative organ weight (data not shown). Anemia was detected in mutants at this early age and could partly account for the liver color alteration. However, the spotted aspect and the presence of large white areas in the hepatic lobes in all *Kit*^*Sco*5/*Sco*5 ^analyzed at 7.5 dpp (n > 10) were associated with an abnormal structure of hepatocytes (Figure 3D). Indeed, lipid microvesicular accumulation, or steatosis, was detected in mutant hepatocytes by specific staining at 7.5 and 10.5 dpp with Oil-red-O (Figure 3D). Similar lipid inclusions were observed in the liver of *Kit*^*Whc*1/*Whc*1 ^neonates (data not shown), to a lesser extent in *Kit*^*Sco*5/+ ^animals and also in few *Kit*^*Sco*5/*Sco*5 ^individuals that survived until two months of age (Figure 3E). A comparable phenotype was detected in 7.5 dpp and adult *Kit*^*W*/*W*-*v *^mice (Figure 3E). By contrast, *Kit*^*Sow*3 ^and *Kit*^*Sco*1 ^homozygotes developed normally and did not display any hepatic phenotype. Thus the liver phenotype was not specific to the *Sco5 *allele of *Kit*, as it was also found in a number of compound *Kit *mutants.

**Figure 4:**
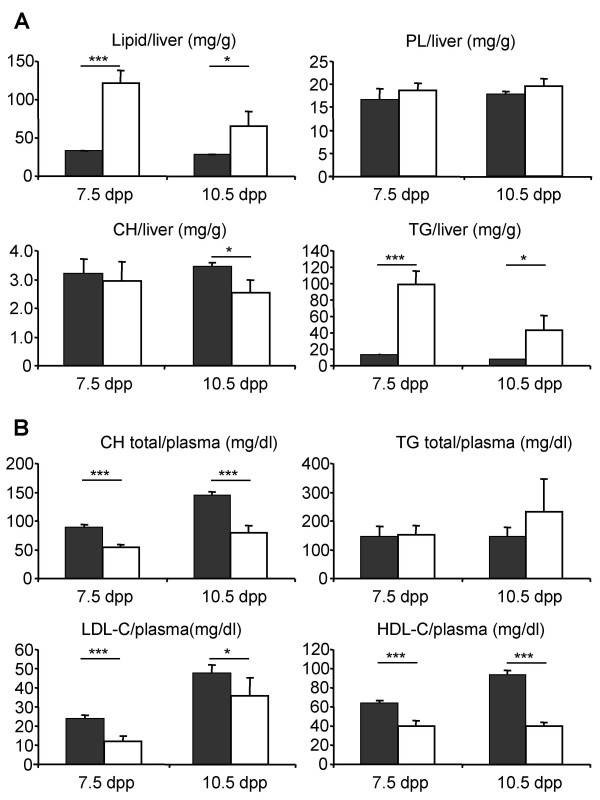
**Quantification of lipids in the liver and plasma of *Sco5/Sco5 *mutants**. Lipids were quantified from the liver (**A**; 5 individuals per genotype) and from the plasma (**B**; 10 individuals per genotype) of mutant and wild-type littermates at 7.5 and 10.5 dpp. Concentrations are indicated as a mean ± s.e.m and expressed as mg/g of tissue or mg/dl respectively. PL, CH, TG for plasma lipids, cholesterol, triglycerides. HDL-C and LDL-C for cholesterol associated respectively with HDL and LDL.

Then, we investigated the onset of the phenotype by following the lipid/water ratio in the liver of newborn mice using nuclear magnetic resonance spectroscopy (NMR). Accumulation of hepatic lipids in *Kit*^*Sco*5/*Sco*5 ^mutants was detected at 2.5 dpp (Figure 3F) suggesting that defect started earlier. Moreover the lipid/water ratio increased until 8.5 dpp when most of the homozygotes begun to die (5 out of 8 studied) as predicted from the original analysis of viability. Nevertheless, the lipid/water ratio was still high in surviving mutants compared to controls at 10.5 dpp (Figure 3F). To further define the onset of the phenotype, liver sections from groups of mutants and control littermates (n = 3) were analyzed during post-natal development. No change was observed immediately after birth in neonates that had not yet suckled any milk (Figure 3G). However, microvesicular accumulations of lipids were found at 1.5 dpp in the liver of wild-type suckling animals from the C3HeB/FeJ, C57BL/6J and 129S2 inbred lines. In the *Sco5/Sco5 *mutant, a similar accumulation of lipids appeared at 1.5 dpp but remained later on, showing that lipids, derived mainly from the milk, were efficiently absorbed by the intestine, transported in the blood but abnormally stored in the liver. The characterization of the hepatic lipid content revealed a dramatic increase of triglycerides with no variation in phospholipids but a slight decrease of the cholesterol in the liver of *Kit*^*Sco*5/*Sco*5 ^mice at 7.5 and 10.5 dpp (Figure 4A). This hepatic accumulation was not followed by an increase of the concentration of plasma triglycerides as observed in obesity or in liver diseases (Figure 4B), but the mutant mice displayed lower concentrations of free and HDL cholesterol as well as LDL cholesterol at both ages. Thus, lipid transport in plasma and exchange between tissues were deficient or not yet mature in *Sco5/Sco5 *suckling neonates, leading to the persistence of the steatosis at 2.5 dpp and later on. Therefore, these results suggest that the lethality induced by *Kit *hypomorphic alleles was concomitant with the impaired lipid metabolism observed in mutant animals during the suckling period.

### *Kit *is expressed in the liver of neonates and adults

**Figure 5:**
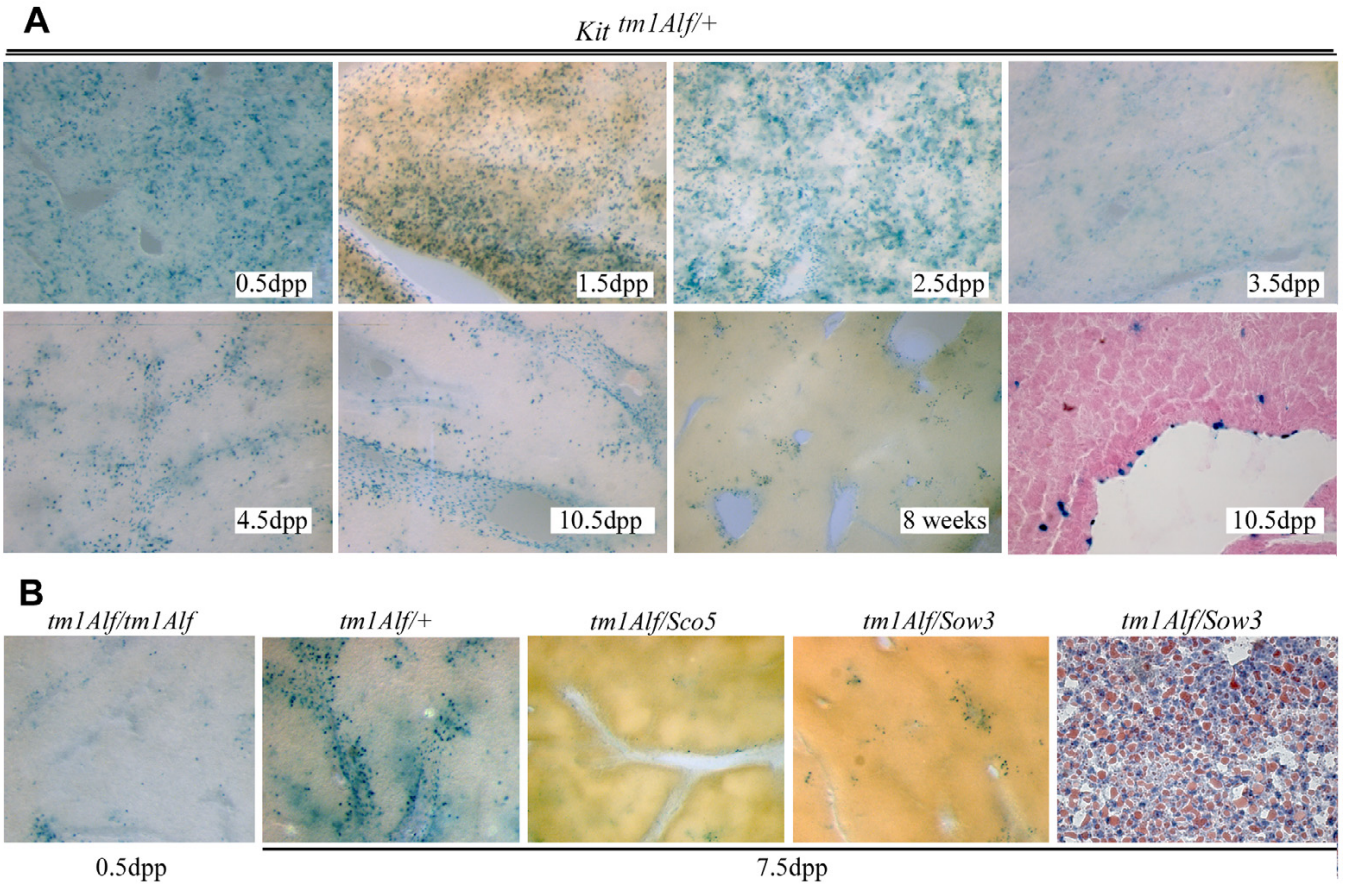
**Hepatic expression of *Kit *correlates with liver phenotype observed in several mutants during the post-natal development and in adult mice**. (**A**) *Kit *expression was detected in the liver of *Kit*^*tm*1*Alf*/+ ^mice by β-galactosidase activity on vibratome sections (40×) from 0.5 to 10.5 dpp, in adult, and on an eosin-stained sections (last panel). From birth to 2.5 dpp, β-galactosidase staining is found in a large number of hepatic cells, a reminiscence of the haematopoietic liver activity that takes place during late stage of embryonic development and ends after birth. As a consequence, the number of dispersed positive cells is reduced at 3.5 dpp. However, Kit expressing cells are clearly observed in the liver from 4.5 dpp to later stages and in adults. In *Kit*^*tm*1*Alf*/*tm*1*Alf *^moribund newborns at 0.5 dpp, β-galactosidase staining is weaker due to the defect in hematopoiesis during fetal development but still remained in few cells. (**B**) At 7.5 dpp *lacZ *expression is detected in *Kit*^*tm*1*Alf*/+ ^liver whereas no expression is observed in transheterozygous *Kit*^*tm*1*Alf*/*Sco*5 ^and *Kit*^*tm*1*Alf*/*Sow*3 ^liver. Such compound heterozygotes suffer from a strong lipid steatosis as shown here for *Kit*^*tm*1*Alf*/*Sow*3 ^liver stained with oil-red-O (last panel). A similar staining was detected in *Kit*^*tm*1*Alf*/*Sco*5 ^liver (not shown).

Consistent with the phenotype, *Kit *gene expression was detected during post-natal development in the liver by using the *lacZ *reporter gene inserted in the first exon of *Kit *from the *Kit*^*tm*1*Alf*/+ ^mouse [8]. *Kit *expression was detected in a large number of cells in the liver from 0.5 to 2.5 dpp, as a reminiscence of fetal hematopoiesis (Figure 5A). Thereafter, *Kit *expression was confined to a few cells located in the hepatic parenchyma and was also found in the epithelial cells of the biliary canals at later stages and in adults. As expected, the number of *Kit*^+ ^cells was dramatically reduced at 0.5 dpp in moribund *Kit*^*tm*1*Alf *^homozygotes [8]. In trans-heterozygotes *Kit*^*tm*1*Alf*/*Sco*5 ^and *Kit*^*tm*1*Alf*/*Sow*3^, a reduced number of *Kit*^+ ^cells were detected at 7.5 dpp while steatosis was observed (Figure 5B and data not shown). Thus, lipid accumulation was induced by several combinations of *Kit *mutations during the post-natal period and its appearance was clearly associated with a decrease in the number of KIT expressing cells in the liver.

### Molecular characterisation of the *Kit*-induced steatosis during post-natal development

**Figure 6:**
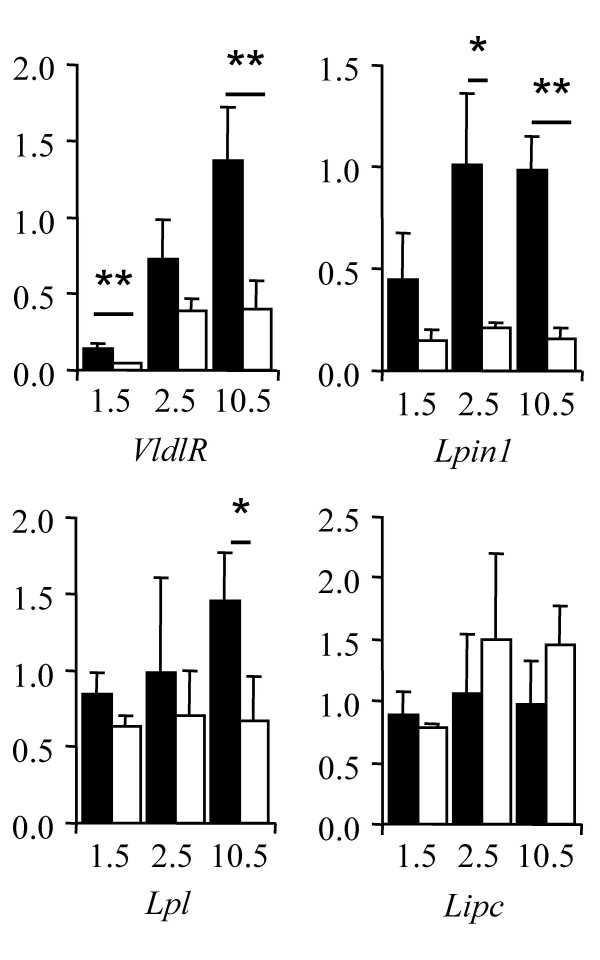
**Misregulation of *VldlR*, *Lipin1 *and *Lpl*, in the liver of *Sco5 *mutants compared to controls**. At 2.5 dpp, a significant dowregulation of *Lpin1 *is observed in mutant (open rectangle) compare to wild type (filled rectangle) while *Vldlr *is significantly downregulated at 1.5 dpp. At 10.5 dpp, the expression level of *VldlR*, *Lipin1 *and *Lpl *is low in mutant compare to wild-type livers whereas the expression of *Lipc *is not affected. All the data were normalized as described in the Materials and Methods and were subjected to statistical analysis (Fischer and Student t tests, * for p < 0.05 and ** for p < 0.01).

To decipher the molecular basis of *Kit*-dependent steatosis, we determined the expression profiles of key genes involved in lipid hepatic metabolism: such as apoliproteins (*Apoa1*, *ApoB*), lipoprotein receptors (*LdlR*, *VldlR*, *Scarb1*, *Lrp1*), lipase (*Lipc*, *LipH*, *Lpl*) and others implicated in hepatic lipidogenesis (*Scap*, *Srebf1*, *Srebf2*), lipid secretion (*Pltp*, *Mttp*, *Abca1*), bile acid synthesis (*Cyp8b1*, *Cyp7a1*), lipid transport (*Slc10a1*, *Abcb11*, *Abcb1a*, *Abcc2*) and a lipodystrophy gene, *Lipin 1 *(*Lpin1*), encoding a phosphatidate phosphatase enzyme with transcription activity [26-28]. Expression of most of the genes remained unchanged in wild-type and mutant individuals at 10.5 dpp (not shown) except for the very low density lipoprotein receptor (*Vldlr*), the *Lpin1 *and the lipoprotein lipase (*Lpl*) genes that were downregulated in *Sco5/Sco5 *mutants compared to wild-type littermates (Figure 6). In addition *Vldlr *expression appeared to be significantly retarded at 1.5 dpp just during the transient steatosis while the expression level reached a plateau at 2.5 dpp with the same expression level found at 10.5 dpp. The downregulation of *Lpin1 *started to be detected at 1.5 dpp and was significant at 2.5 dpp when homozygote *Sco5 *mutants suffer from the clearance of microvesicular accumulated lipids. Interestingly, the *Lpic *gene transcription is not significantly affected in the *Sco5 *mutant at these ages. Altogether, these changes at the transcriptional level of key genes involved in hepatic lipid uptake (*VldlR*) or modification (*Lpin1*, *Lpl*) are helpful to understand the molecular mechanism of the *Kit*-induced defect in the liver.

## Discussion

### A new allelic series of *Kit *as a tool for deciphering downstream signaling pathways

In this paper, we described three new point mutations inducing loss-of-function of the KIT protein (*Sco1*, *Sco5 *and *Whc1*) and one potential mutation affecting the regulation of the *Kit *gene (*Sow3*). Several other mutants affecting the level of *Kit *expression have been described in the literature [2,19,23,29], unraveling that quiet a few dispersed regulatory elements control *Kit *gene expression. Most of them correspond to large chromosomal rearrangement whereas *Sow3 *is presumably a point mutation induced by ENU affecting a regulatory element controlling *Kit *during melanogenesis and gametogenesis. The missense mutations described in *Sco1 *and *Sco5*, at position M623 and V667 respectively, are found in the amino-terminal lobe near the ATP-binding site, whereas for *Whc1*, F809 is located in the DFG motif of the Mg^2+ ^binding loop in the activation segment of the tyrosine kinase domain[14,30]. These residues are highly conserved in the KIT sequence deduced from various species, from human to zebrafish, and also in other mouse receptor tyrosine kinases from the same subfamily such as CSFR1, PDGFR, VEGFR, and FGFR (data not shown). Interestingly, the two residues mutated in *Sco5 *and *Sco1 *are located in the amino-terminal lobe composed of β-strands, nearby the T660, that is mutated in the *W-v *allele, in the structure of the active KIT kinase [13] (Figure 1C). Overall the mutations described here are presumably hypomorphic because complete loss-of-function such as in the *lacZ *knock-in or in the *W *allele are homozygous lethal [8,31]. Of particular interest is the consequence of the F809L mutation located in the activation loop. This region of the tyrosine kinase domain undergoes a conformational change after the transphosphorylation of the autoinhibitory JM domain after KITL binding. Such a change will leave open the ATP-binding site and the Mg^2+ ^binding loop for the receptor kinase activity. In addition, the anti-cancer drug STI-571 interacts notably with the F809 and blocks the tyrosine kinase domain in an inactive conformation [14]. Remarkably, the phenotypes observed in the *Whc1 *homozygotes are similar to the *Sco5 *allele with a strong impact on pigmentation and peripheral blood cells but not on germ cells. The four *Kit *mutants display pleiotropic phenotypes affecting with variable degrees the melanogenesis, hematopoiesis and gametogenesis in homozygotes. Interestingly, *Sco1*, *Sco5 *and *Whc1 *displayed essentially normal gametogenesis suggesting that the M623, V667 in the ATP binding site and the F809 in the activation loop domain are not as important as the Y719 bound by the phosphatidylinositol 3'-kinase (PI3K) that is essential for *Kit *function in testis [20]. Therefore the model of the inhibitory effect of the JM domain should be reconsidered for the germ cells to reconcile the absence of major germline defect in the *Whc1 *mutant. These data stress out the complexity of the downstream signaling pathways and the targets that are controlled by the KIT protein in different organs.

### The impaired viability of *Kit *mutants is associated with an altered hepatic lipid metabolism

Similar to the *Kit*^*W*/*Wv *^and the *Kit*^*Wads*/*Wads *^mutants recently described [18], the post-natal viability of the *Whc1 *and of the *Sco5 *homozygotes is strongly affected. Further detailed analysis identifies a major defect in hepatic lipid metabolism with steatosis at 7.5 dpp in animals carrying various combinations of hypomorphic *Kit *alleles, including *W/W-v, Sco5/lac, Sow3/lac, Sco5/Sco5 *and *Whc1/Whc1*. Furthermore the appearance of steatosis is correlated with the defect in growth of the mutant initiated at 3.5 dpp. The determination of hepatic lipid content correlated the steatosis with a deregulated accumulation of TG just after suckling. During mammalian post-natal development, TG originate predominantly from the maternal milk and represent the major source of energy available for the neonates [32-34]. Ingested TG are assembled in chylomicrons particles and delivered to the developing organs. Following the local action of Lipoprotein Lipase (LPL), TG-derived free fatty acids (FFA) are absorbed by tissues while the remaining of the chylomicrons, the remnants, is taken-up by the liver. During the fasting period, the liver participates in the uptake, oxidation, de novo synthesis, assembly and secretion of TG in very low density lipoproteins particules (VLDL; [35]. TG in VLDL are then used by the tissues depending on their LPL activity. The abnormal level of circulating LDL-C observed in *Kit *mutants suggests a defect in the liver to export and metabolize lipids from maternal milk. In addition, the maintenance of the "transient steatosis" state in the *Kit *mutants, observed in wild-type animals in the initial suckling phase just before the third post-natal day (this work; [36]. We suggest that the transient steatosis observed in wild-type animals corresponds to a transitory accumulation of lipids while the liver organ of neonates adapts its physiology to the triglyceride-rich regime of the suckling period. This was further supported by the expression analysis of genes such as *VldlR *and *Lpin1 *that start to be expressed at 2.5 dpp (this study). In *Kit *mutants, a defective hepatic metabolism, with the abnormal low expression of *VldlR *and *Lpin1*, leads to the maintenance of this "transient steatosis".

Expression profiling for key genes participating to the metabolism, the transport, the modification, lipidogenesis and biliary acid synthesis in the liver revealed that only a few genes, namely *Vldlr*, *Lpin1 *and *Lpl *were downregulated in *Kit *mutants. Consistent with the *Kit*-induced hepatic phenotype, interference with lipase function induces type I hyperlipoproteinemia in human (OMIN238600) and more severe hyperchylomicronemia affecting post-natal viability in *Lpl *mutant mice [37] or in *Combined Lipase deficiency *(*Cld*), which alters both *Lpl *and Hepatic lipase (*Lipc*) activities [38-40]. In addition, VLDLR plays a major role in the metabolism of postprandial lipoproteins by enhancing LPL-mediated TG hydrolysis [41] and the *Vldlr *mutant mice suffer from growth retardation during the suckling period [37]. *Lpin1 *reduced expression is presumably a major event leading to the abnormal metabolism of lipid in *Kit*^*Sco*5/*Sco*5 ^mice. Indeed, *Lpin1 *loss-of-function induces steatosis and juvenile lethality in the "*fatty liver dystrophy*" (*fld*) mice, a model of human lipodystrophy [27,42]. Recently, *Lpin1 *has been shown to complement the phosphatidate phosphatase yeast deficiency [28]. In yeast its homolog regulates the level of triacylglycerol and FFA and represses the expression of genes involved in phospholipid synthesis. Similarly the hepatic fatty acid oxidation is reduced in *fld/fld *mice [43] and *Lpin1 *is regulated by the transcription factor PGC-1α that regulates several metabolic pathways. In addition LIPIN1 can activate the transcription of *Pparα *and coactivates with PGC-1α and PPARα a large panel of other genes controlling lipid metabolism [44]. Therefore, downregulation of *Lpin1 *in *Kit *mutants will lead to a reduced oxidation of lipids and steatosis in a context of increased lipid delivery during suckling. Similar to *fld/fld *mice, some *Kit*^*Sco*5/*Sco*5 ^animals could overcome steatosis after weaning but the most affected ones continued to display it. Consistent with our data, hyperlipidemia was observed in a fraction of adult *Kit*^*W*/*W*-*v *^mice and correlated with an abnormal plasmatic lipoprotein profile and reduced lipolytic activities [45]. In addition to the *Lpin1 *downregulation, repression of *Lpl *and *Vldlr *would also contribute to the hepatic *Kit*-induced phenotype. Interestingly, *Lpl *homozygous mutants died within 48 h after birth with severe hypertriglyceridemia [37]. Thus, linked to the steatosis observed in *Kit *hypomorphic mutants were changes in the expression of genes that are known to induce hepatic lipid metabolism anomalies in mutant mice. Such a change might explain why the hepatic deficiency is found in the surviving *Kit *homozygote adults. The absence of Kit expressing-cells and/or reduced Kit activity during development might have an indirect effect. In such a way, *Kit *loss-of-function mimicked the downregulation of *Lpin1*, *Lpl *and *Vldlr *leading to juvenile steatosis and growth retardation. Consistently, KIT and KITL were found to be expressed in the rat cholangiocytes [9] and several mouse mutants of *Kitl *displayed comparable low post-natal viability [46,47].

### Involvement of the *Kit*-dependent steatosis and anemia in post-natal lethality

The origin of the post-natal deficiency observed in *Kit *mutants is commonly associated with anemia. The mutant post-natal phenotype is improved by the injection of mouse embryonic liver cells or bone marrow derived cells, 24 h after birth, respectively in 26% and 32% of *W/W-v *mutants [48]. The rescue is even better if the transplantation of embryonic liver cells is done through the placental circulation by day 11 of gestation [49]. Similarly the rescue of *Kit *mutant mice could be achieved by erythropoietin [50]. Interestingly in such rescue, the RBC counts and the percentage of homozygous mutants that survived the post-natal crisis are similar to our results confirming that another KIT-dependent factor influences the survival of the pups after birth. Thus we propose that the liver defect induced by Kit partial loss-of-function is one of these additional factors.

We described some molecular events affecting the neonatal hepatic function, but other changes in the liver of *Kit *mutants can not be ruled out. Additional analysis will help to describe in details the cellular mechanisms underlying the liver phenotype induced by *Kit *loss-of-function. Thus, even though *Kit*-induced anemia might participate to the post-natal crisis observed in the mutant mice, we propose that *Kit*-induced steatosis is a major factor at the origin of the post-natal mortality. Only a controlled inactivation of *Kit *in blood or liver compartments will help to clearly discriminate the impact of anemia and liver defect in the *Kit*-dependent impaired post-natal viability.

### KIT and the signaling pathways controlling lipid metabolism in the liver

KIT is a receptor protein kinase controlling several signaling pathways. Some of them are known to control liver metabolism, such as the PI3K-AKT-mTOR, JAK/STAT [51,52], or to induce growth defect like for example in the *Snai2 *mutant [12,53]. Thus another alternative to explain the importance of KIT in liver homeostasis could be due to the alteration of signaling pathways leading to impaired lipid metabolism. Alternatively, KIT could participate directly to the signaling pathway controlled by lipoprotein receptors. PDGFRB, another member of the same receptor family, is a co-receptor of the LDL receptor related protein [54,55]. Similarly, KIT could cooperate with members of the lipoprotein receptors family, such as VLDLR, or could control the expression of *Lpin1*, regulating the hepatic triglyceride metabolism and oxidative phosphorylation through its key position in the PPARa/PGC-1a regulatory pathway [44]. Interestingly, altering the binding of PI3K with the KIT protein, and reducing the activation of AKT to 10%, does not affect the weight of mutant animals [20], suggesting that, during the post-natal development, the KIT-dependent metabolism of triglycerides is not fully associated with the PI3K pathway but results from another signalling pathway.

### Kit and the Fetal liver stem cells

KIT is a cellular marker of fetal liver stem cells, is involved in the differentiation/proliferation of the bipotent hepato-biliary progenitor cell [56,57]. In addition Kit expression is used to isolate population of hepatocytes during *in vitro *differentiation of ES cells [58]. Consistently, KITL is an important factor controlling hepatocyte proliferation after hepatectomy in IL6 mutant mice [59]. Thus, the steatosis could be an indirect consequence of a defect in the differentiation or the proliferation of hepatic progenitor cells leading to the loss of KIT+ cells observed in *Sco5/lac *or *Sow3/lac *animals. Decreasing the activity of KIT to a certain steatosis threshold will modify the pool of stem cells that will affect either the development of the liver and the post-natal hepatic homeostasis. Consequently, the defect in lipid metabolism of *Sco5/Sco5 *adults might result from a consequence of a post-natal defect that was not compensated during the juvenile development. This hypothesis is in agreement with the pattern of expression of the genes tested. Kit expression is restricted in the juvenile and adult liver. Thus we should foresee an indirect effect of Kit on hepatocytes functions observed in this study.

## Conclusion

Taken together, these data demonstrate the key function of *Kit *in neonates liver physiology and further explain the early hepatic defect induced by *Kit *loss-of-function. Now, KIT should be considered as one of the endogenous factors that could lead to steatosis [60]. A very low number of animal models are available to study the consequences of impaired lipid metabolism during suckling, reminiscent of some human conditions (OMIN 212140, 228100). These data open new perspectives for the molecular understanding of juvenile steatosis, the analysis of Kit function during liver development and support the need to follow-up hepatic function in patients during anti-cancer treatment with the inhibitor of KIT activity, STI-571, reported to affect the weight and the viability of rat neonates [61].

## Methods

### Mice

The *Sco1, Sco5, Sow3 *mutant alleles were isolated from the chemical mutagenesis program ongoing in the Institute of Experimental Genetic at the GSF (Neuherberg, Germany) whereas the *Whc1 *was recovered in a similar mutagenesis screen in Orleans. Investigations were achieved in the same genetic background C3HeB/FeJ (C3Fe) for the phenotypic analysis. Outcross and backcross on C57BL/6 (B6) were performed for the genetic mapping except for *Whc1 *which was generated in a mixed C3Fe.B6 background. The B6 *Kit*^*W *^and *Kit*^*W*-*v *^alleles obtained from the TJL (Bar Harbor, Maine, USA) carry respectively a deletion of the transmembrane domain and a missense mutation at T660 M. The 129S2 *Kit*^*tm*1*Alf *^mouse line corresponds to the disruption of the *Kit *gene sequence due to the insertion of a *lacZ *reporter gene in the first exon [8]. Standard diet (SDS) and water were provided ad libitum. All the mice were bred under SPF conditions and experiments were carried out according to the guidelines of the French Ministry of Agriculture for experiments with laboratory animals (law 87848 and YH accreditation 45–31).

### Genetic and comparative gene sequence analysis

A panel of 60 MIT markers polymorphic between C3Fe and B6 was used for a genome-wide linkage analysis as described previously [21]. For complementation analysis, heterozygous mutant mice carrying the *Sco5, Sco1, Sow3 *or the *Whc1 *alleles were mated separately with *Kit*^*W*^, *Kit*^*W*-*v *^and *Kit*^*tm*1*Alf *^heterozygous animals. Animals with a white coat color were classified as trans-heterozygous individuals.

For the sequence determination and the genomic DNA analysis, specific primer pairs were designed in the flanking sequences of each exon, except for the large exon 21, for which a series of overlapping PCR products were generated (Primer sequence available upon request). The amplicons generated from DNA isolated from two littermates corresponding to each phenotype (wild-type, heterozygous or homozygous mutants) were directly sequenced using the Big-dye terminator kit and analyzed on an ABI 310 automated sequencer. The nucleotide changes noticed in *Sco1*, *Sco5 *and *Whc1 *alleles were confirmed to be specific to the mutant lines and not found in the founder strain C3HeB/Fe or C57BL/6J and others control strains including DBA, CBA, BALB/c, RIII/Dmob, RIIIS/J, 129, A/J, AKR, PWK, IS/CamRk, IS/Cam Ei, PWD, Cast, NOD.

### Phenotypic analysis

Groups of age- and sex-matched mutant and wild-type littermate mice were subjected to a series of tests following the recommendations from the standard operating procedures developed inside the EUMORPHIA network [62]. Several parameters were automatically scored for blood cell analysis by taking 100 μl of blood from suborbital sinus of 6–8 week-old anaesthetized mice (isoflurane), collecting them in EDTA tubes (VWR) and running them through a Technicon H1 hematology analyser (BAYER). Fertility tests were carried out for a period of 4 months using 5 homozygous males mated with two wild-type females from the same genetic background. Females were checked for pregnancy every week and changed every two months.

For histological analysis, the liver was fixed overnight in Bouin's solution, embedded in paraffin wax and subjected to classical procedures for hematoxylin-eosin (H&E) staining. Frozen tissue sections were post-fixed with formalin solution (Accustain, Sigma) before staining with Oil-red-O (ORO) and hematoxylin. Sections were observed under a light microscope (Leica). For *lacZ *staining, sections of whole fresh liver were made on a vibratome or a cryotome (Leica) and post-fixed with 5% paraformaldehyde. The β-galactosidase activity was revealed using a standard procedure [63]. Pictures were taken with a Leica digital camera and processed with the Adobe Photoshop (v.7) software.

The *in vivo *NMR experiments were carried out on a 9.4 T horizontal imaging spectrometer (Bruker Biospec, USR, Wissembourg, France) equipped with a 950 mT/m gradient coil and a 20 mm inner diameter 1H custom made surface coil. The mice were anesthetized with 1% isoflurane (TEM, Bordeaux) and the animal's temperature and respiratory signal were controlled during anesthesia. After liver imaging, for spectroscopy, 1D 1H PRESS spectra were recorded on 2 × 2 × 2 mm^3 ^voxels located inside the liver (TR = 4 s, TE = 12 ms, na = 64, acquisition time = 6 min) after manual or semi automatic shimming. The total duration time was about 45 min per mouse. These spectra were used to quantify the lipids inside the liver compared with the water signal. The water and lipids peaks areas were integrated using the Xwinnmr Bruker software.

The lipid composition of the liver was evaluated using the method described in Boehler et al. [64]. Briefly, frozen livers were thawed and homogenized in propanol-2 using an ultra-turrax apparatus (Janke & Kunkel Gmb, Staufen, Germany). After incubation at 60°C for 1 h and centrifugation for 5 min at 3000 g, the supernatant was collected and the pellet was re-extracted with propanol-2. Phospholipids, triglycerides, and total cholesterol were measured enzymatically on the pooled propanolic extracts using appropriate kits: triglycerides PAP 150, phospholipids PAP 150 and Cholesterol RTU (Biomérieux S.A., 69280 Marcy l'étoile, France). Blood chemistry analysis was carried out with plasma isolated from Li-heparin-treated blood on an Olympus AU400 autoanalyzer (Olympus, Hamburg, Germany).

### Expression analysis by quantitative RT-PCR

Total RNA was extracted from the liver using Trizol reagent (Life Technologies). Random hexamers were used for priming the first cDNA strand synthesis with 1 μg of total RNA. We tested the expression of a panel of genes involved in hepatic lipid metabolism and known to be expressed in hepatocytes (*LdlR*, *Lrp1*, [65], ApoA1 [66], ApoB [67], *Cyp8b1*, *Cyp7a1 *[68], Abca1 [65], *Ntcp*, *Scap*, *Srebf1*, *Srebf2 *[69,70]), in canicular membrane (*Abcb11*, *Abcb1a*, *Abcc2*, *Abcb4 *[65,69,70]), in both cell type (*Scarb1 *[71,72], Abcb4 [65,69]), in Kupffer cells (*Lpl *[73]) or in a non specified hepatic area (*LipH *[74]; *VldlR *[75], *Pltp *[76], *Mttp *[77], *Lipin1 *[27]). The gene specific primer pairs for *Ldlr*, *Vldlr*, *ApoeR*, *Lpdl*, *Lpin1*, *ApoB*, *Mttp*, *Abca1*, *Scap*, *Srebf1*, *Srebf2*, *Cyp7a1*, *Abcc2*, *β2-microglobulin *were obtained from Quiagen (France) and the others from Primerbank [78]. They were used to amplify the specific transcripts from cDNA obtained from at least five different individuals, and quantified using Sybergreen on MX4000 apparatus (Stratagene, La Jolla, USA) using the 18S and β2m RNAs as internal standards. Data were normalized for each gene using the 2^-ΔΔCt ^method in controls and *Sco5 *homozygotes littermates (n > 5 at 10.5 dpp or n > 4 at 2.5 dpp per group) after standardization with the 18S rRNA and β2-m mRNA.

### Statistical analysis

All the results are expressed by the mean ± s.e.m (standard error of the mean). Statistical analysis was carried using the Fischer's test and the Student's t-test and the null hypothesis was rejected for P < 0.05 unless otherwise indicated.

## Abbreviations

ENU: *N*-Ethyl-*N*-NitrosoUrea

C3Fe: C3HeB/FeJ

B6: C57BL/6J

NMR: Nucleic Magnetic Resonance

## Authors' contributions

LM conceived and performed most of the experiments and did the histological analysis for the *Sco5*, *Sco1 *and *Sow3*. MCC collected the samples and did the QRT-PCR analysis on the liver of mutant and control mice. VN and SA generated the genetic mapping of the *Sco5*, *Sco1*, *Sow3 *and *Whc1*. SR performed the post-natal survey and collected the samples for blood analysis. HF isolated and established the Sco5, Sco1 and Sow3 mutant lines MK did the blood chemistry analysis. PP analysed the samples from the Whc1 mutant. MR performed the lipid analysis of the liver. BTD and JCB performed the MRI experiments. JJP collected the data for the expression analysis in the liver. AP controlled the Kit sequence polymorphism in several mouse lines. MH helped draft the manuscript. YH drafted the final manuscript. All authors read and approved the final manuscript.
